# Barriers to health insurance uptake in Rwanda: a nationwide cross-sectional survey

**DOI:** 10.11604/pamj.2025.51.8.45920

**Published:** 2025-05-08

**Authors:** Roger Muremyi, Leon Benoit Migisha, Marx Louis Pasteur Munezero, Shallon Mumararungu, Ebenezer Niyigena, Gasafari Willy Mpabuka, Jennifer Batamuliza, Charles Ruranga

**Affiliations:** 1 Department of Applied Statistics, University of Rwanda, Kigali, Rwanda,; 2 Department of Information Technology, University of Rwanda, Kigali, Rwanda,; 3 African Centre of Excellence in Data Science, University of Rwanda, Kigali, Rwanda

**Keywords:** Health insurance, community-based health insurance, universal health coverage, total health expenditure, Rwanda

## Abstract

**Introduction:**

achieving universal health coverage by 2030 is a key objective for Rwanda, ensuring equitable access to health insurance. However, approximately 10% of the population remains uninsured. This study aims to identify socio-economic and demographic factors associated with health insurance uptake in Rwanda.

**Methods:**

we analyzed secondary data from the Fifth Integrated Household Living Conditions Survey (EICV 5), comprising 14,580 households. Multivariable logistic regression was performed to assess factors associated with health insurance uptake. Descriptive statistics were used to summarize the characteristics of the surveyed households.

**Results:**

the study population had a mean household size of 4.8 individuals, with 62% residing in rural areas. Among household heads, 56% were male, 44% were female, 38% had no formal education, and 64% were employed. Multivariable logistic regression analysis revealed that household heads in the highest wealth quantile had higher odds of being insured (aOR: 3.82, 95% CI: 3.37-4.33; p < 0.001) compared to those in the lowest quintile. Those with no formal education had lower odds of being insured (aOR: 0.57, 95% CI: 0.46-0.71; p < 0.001) than those with higher education. Residents of Kigali City had greater odds of being insured (aOR: 1.52, 95% CI: 1.31-1.75; p < 0.001) compared to residents in other regions. Females were more likely to be insured than males (aOR: 1.21, 95% CI: 1.11-1.34; p < 0.001), while single household heads had lower odds of insurance uptake (aOR: 0.61, 95% CI: 0.60-0.74; p < 0.001) compared to married counterparts. Younger individuals were less likely to be insured (aOR: 0.32, 95% CI: 0.29-0.49; p < 0.001) compared to older adults.

**Conclusion:**

health insurance uptake in Rwanda is significantly influenced by socio-economic and demographic factors, including wealth status, education level, geographic location, gender, marital status, and age. Targeted interventions should prioritize vulnerable groups, such as low-income and less-educated individuals, young adults, and rural residents, to improve insurance coverage.

## Introduction

Health insurance is a financial mechanism designed to protect individuals from the high costs of medical care by providing coverage for healthcare expenses. It plays a crucial role in ensuring access to essential health services, reducing out-of-pocket payments, and preventing financial hardship [1]. Achieving universal health coverage (UHC) remains a global priority, with the World Health Organization emphasizing the need for equitable healthcare access [2]. In many African countries, a significant portion of the population struggles with limited healthcare access due to financial constraints. Many households experience complete or partial exclusion from healthcare services, often leading to catastrophic health expenditures and poverty [3]. Socioeconomic factors such as income, education level, and employment status significantly influence health insurance uptake. Research indicates that individuals with higher income levels and stable employment are more likely to be insured, whereas low-income groups often face affordability challenges [4]. Economic constraints can prevent individuals and families from affording health insurance premiums, deductibles, and co-payments, thereby limiting their ability to enroll in comprehensive health insurance plans [4]. Furthermore, a lack of awareness about available insurance schemes further hinders enrollment, especially among rural and marginalized populations [5]. Community-Based Health Insurance (CBHI) has emerged as a promising model for improving healthcare access in low-income settings [6]. Rwanda has been a leader in implementing CBHI, incorporating it into its national health strategy [7]. The CBHI scheme, structured under the Ubudehe categorization system, allows households to contribute premiums based on their income levels [8]. This approach has significantly expanded insurance coverage, ensuring that vulnerable populations receive subsidized healthcare services. As a result, over 90% of Rwandans are now covered under some form of health insurance [9]. However, the remaining uninsured population-approximately 10% continues to face barriers in accessing healthcare services [10]. Despite Rwanda´s progress, achieving full UHC remains an ongoing challenge. Population growth, economic shifts, and evolving healthcare needs require continuous policy adaptation to ensure sustainability and inclusivity [11]. Understanding the factors influencing health insurance uptake is essential for designing targeted interventions to close the remaining coverage gap. This study aimed to identify the socio-economic and demographic factors associated with health insurance uptake in Rwanda, using data from the Fifth Integrated Household Living Conditions Survey (EICV 5).

## Methods

**Study design and setting:** this study utilized secondary data from the Fifth Integrated Household Living Conditions Survey (EICV 5) conducted in 2016-2017 [12]. The survey was carried out by the National Institute of Statistics of Rwanda (NISR) and covered all administrative regions of Rwanda. The EICV 5 survey provides nationally representative data on various socio-economic indicators, including poverty, employment, education, and healthcare access [13].

**Study population:** the study population consisted of households sampled in EICV 5, which included 1,260 villages and 14580 households [14]. The target population comprised household heads whose health insurance status was recorded in the dataset. Inclusion criteria required households with complete information on health insurance enrollment and socio-economic characteristics. Households with missing or incomplete responses regarding health insurance status were excluded. The sample size was determined using a multistage stratified random sampling technique, ensuring national representation [15].

**Data collection:** the EICV 5 survey data were collected by NISR using structured questionnaires administered through face-to-face interviews [16]. Data collection followed a multistage random sampling method, based on the 2012 Rwandan Census, with 3,960 villages selected to represent the country in various national household surveys, including EICV 5 [16]. The dataset included detailed information on household demographics, socio-economic status, and health-related factors [17].

**Definitions:** the dependent variable in this study was health insurance uptake, defined as a binary variable: 1 if the household head had health insurance and 0 otherwise [18]. Independent variables included demographic and socio-economic factors such as age group, sex, poverty category, wealth quintile, Ubudehe category, geographic region, education level, disability status, marital status, and yearly wages [19].

**Statistical analysis:** data analysis was conducted using Stata software [20]. Descriptive statistics were used to summarize the characteristics of the study population. Unvariable and multivariable logistic regression analyses were performed to assess factors associated with health insurance uptake [21]. Variables with a p-value <0.05 in the unvariable analysis were considered for inclusion in the multivariable model. The logistic regression model used was:


$$In\left(\frac{p}{1-p}\right)=\beta_{0}+\beta_{1}x_{1}+\beta_{2}x_{2}+\beta_{3}x_{3}+\beta_{n}x_{n}$$

P: this represents the probability of success or the probability that the head of household has health insurance, 1-P: this represents the probability of failure or the probability that the head of household does not have health insurance, β_0_: this represents the constant, x_1_… x_n_: represent the independent variables, β_1_…β_n_: represent the coefficients.

**Ethical considerations:** ethical approval for the study was granted by the relevant institutional review board [22]. Since this study used publicly available secondary data from NISR, informed consent was obtained at the time of data collection by the original survey administrators [23]. The dataset used was anonymized to ensure confidentiality and privacy of the respondents [24].

## Results

**General characteristics of the study population:** Table 1 presents the general characteristics of the study population. Among the heads of household participants, 75.25% have health insurance, while 24.75% do not. The majority of the participants are male (74.46%), with females representing 25.54%. The age distribution shows that households headed by individuals over 76 years comprise 4.54%, while those aged 14-24 and 66-75 represent 4.92% and 6.92%, respectively. The highest proportion (29.02%) belongs to the age group 25-35. Educational attainment reveals that 68.91% of heads of households have only primary education, 20.66% have secondary education, and 10.43% are university graduates. Regarding economic status, 66.82% of households are categorized as non-poor, while 20.1% are moderately poor and 13.07% are severely poor. Most heads of households (73.04%) fall into the higher wage category, and 40.97% belong to Ubudehe category 3. Most households are located outside Kigali city (88.89%), and 91.56% do not have a disability. The findings from Table 2 indicate the distribution of health insurance coverage among respondents indicates that the vast majority are enrolled in mutual insurance schemes, with 69.75% of the total population covered under this category. Among the insured respondents, 92.26% (10,169 out of 10,972) rely on mutual insurance, highlighting its central role in Rwanda´s health financing landscape. A smaller proportion of insured individuals are covered by RAMA (3.55%), other insurance types (0.92%), MMI (0.88%), and employer-based schemes (0.16%). Notably, none of the uninsured respondents are covered by any type of health insurance, with all 3,608 reporting no insurance at representing 24.75% of the total sample. This underscores the importance of mutual insurance as the dominant form of health coverage in Rwanda and highlights the gap that still exists in reaching universal health coverage, especially for those currently not enrolled in any scheme.

**Table 1:** insurance coverage by gender

Covered by health insurance		Sex		
		Female	Male	Total
No	Number	914	2,694	3,608
	%	24.54	24.82	24.75
Yes	Number	2,810	8,162	10,972
	%	75.46	75.18	75.25
Total	Number	3,724	10,856	14,580
	%	100.00	100.00	100.00

**Table 2:** insured respondents by the type of health insurance

Insured respondent		Name of the main type of health insurance						
		Employer	MMI	Mutual insurance	None	Other insurance	RAMA	Total
No	Number	0	0	0	3,608	0	0	3,608
	%	0.00	0.00	0.00	24.75	0.00	0.00	24.75
Yes	Number	23	128	10,169	0	134	518	10,972
	%	0.16	0.88	69.75	0.00	0.92	3.55	75.25
Total	Number	23	128	10,169	3,608	134	518	14,580
	%	0.16	0.88	69.75	24.75	0.92	3.55	100.00

**Factors associated with health insurance coverage:** the univariable analysis revealed several significant predictors of health insurance uptake (Table 3). Factors associated with higher health insurance coverage include being female (aOR: 1.20, 95% CI: 1.01-1.43; p = 0.04), attaining higher education levels (aOR: 0.75 for primary vs. university; aOR: 0.49 for secondary vs. university), and belonging to higher Ubudehe categories (aOR: 7.20 for category 1; aOR: 3.40 for category 4; aOR: 3.20 for category 3; aOR: 1.80 for category 2). Age also plays a significant role, with those aged 14-24 being less likely to be insured (aOR: 0.30), followed by those aged 46-55 (aOR: 0.53). Wealth quintiles further demonstrate a strong correlation with health insurance uptake, where heads of households in the highest quintile are more likely to be insured (aOR: 3.80), and those in lower quintiles are less likely (aOR: 1.20 for quintile 2). Additionally, residing in Kigali city increases the likelihood of being insured (aOR: 1.50). Marital status also influences health insurance uptake; widows are more likely to have insurance (aOR: 1.20), while singles are less likely (aOR: 0.60) compared to married individuals.

**Table 3:** univariable analysis and multivariable logistic regression model

Factor	Categories	Number of households	Percent	Cum.	Odds ratio (95% CI)	P-value
**Insurance coverage**	No	3,608	24.75	24.75	-	-
	Yes	10,972	75.25	100	-	-
**Sex**	Male	10,856	74.46	100	1.22 (1.11-1.34)	0
	Female	3,724	25.54	25.54	-	-
**Age**	between 14-24 years	717	4.92	100	-	-
	between 25-35 years	4,231	29.02	54.66	0.54 (0.46-0.71)	0
	between 36-45 years	3,291	22.57	77.24	0.57 (0.46-0.71)	0
	between 46-55 years	2,602	17.85	95.08	0.54 (0.43-0.67)	0
	between 56-65 years	2,068	14.18	25.64	0.38 (0.29-0.49)	0
	between 66-75 years	1,009	6.92	11.46	0.76 (0.60-0.96)	0.019
	above 76+	662	4.54	4.54	0.90 (0.69-1.17)	0.424
**Education attended**	Primary	3,556	68.91	100		
	Secondary	1,066	20.66	31.09	0.75 (0.68-0.83)	0
	University	538	10.43	10.43	0.49 (0.40-0.61)	0
**Poverty**	Non-Poor	9,743	66.82	66.82		
	Moderately Poor	2,931	20.1	86.93	0.96 (0.72-1.28)	0.779
	Severely Poor	1,906	13.07	100	1.04 (0.72-1.49)	0.841
**Wages**	Lower wages	2,961	20.31	100		
	Middle wages	970	6.65	6.65	0.62 (0.52-0.74)	0
	Higher wages	10,649	73.04	79.69	0.75 (0.63-0.88)	0.093
**Ubudehe categories**	Not found on the list	1,379	9.46	100		
	Category 1	2,318	15.9	15.9	1.89 (1.67-2.13)	0
	Category 2	4,879	33.46	90.54	3.21 (2.84-3.63)	0
	Category 3	5,973	40.97	57.08	3.48 (1.42-8.55)	0.006
	Category 4	31	0.21	16.11	7.21 (6.08-8.55)	0
**Marital status**	Married					
	Divorced				0.67 (0.60-0.74)	0
	Single				1.20 (1.08-1.34)	0.001
	Widow				0.80 (0.47-1.34)	0.394
**Region**	Kigali city				1.52 (1.31-1.75)	0
	Other region					
**Disability**	Yes				1.16 (1.00-1.35)	0.05
	No					-
**Constant**	-	-	-	-	0.68 (0.42-1.09)	0.112

## Discussion

The aim of this research was to analyze the factors that hinder the uptake of health insurance in Rwanda. Our findings reveal that a complex interplay of socio-economic, demographic, and geographic factors significantly influences whether households secure health insurance. Understanding these determinants is critical for developing targeted policy interventions to achieve universal health coverage in Rwanda. One of the most striking findings is the significant gender disparity in insurance uptake. The results indicate that female heads of households are more likely to have health insurance compared to their male counterparts, with an adjusted odds ratio (aOR) of 1.20. This observation aligns with previous studies that have documented similar trends among informal sector workers in Nigeria [25]. A plausible explanation for this gender difference is that females generally exhibit a greater need for healthcare services, particularly about maternal and reproductive health. Consequently, women may be more motivated to secure insurance to mitigate the financial risks associated with childbirth and other health-related issues. However, this trend also suggests that men might be underestimating their health risks or are less aware of the long-term benefits of having health insurance. Therefore, targeted public health campaigns and education programs that emphasize the importance of insurance for all genders could help bridge this gap and improve overall coverage. Education is another critical determinant influencing health insurance uptake. Our study shows that households with lower education levels are significantly less likely to be insured, with an aOR of 0.75 for those with only primary education compared to university graduates. This finding is consistent with the work of Liu K *et al*. [26], who underscored the importance of education in shaping health-seeking behaviors. Individuals with higher educational attainment are more likely to understand the benefits of health insurance, navigate the complexities of insurance enrollment, and appreciate the importance of financial planning for healthcare needs. In contrast, those with limited education may be less aware of available insurance options or may not fully grasp the concept of risk pooling.

These insights highlight the necessity of enhancing health literacy through educational initiatives, which could empower citizens to make informed decisions about health insurance. Socio-economic status, as measured by both poverty levels and the Ubudehe categorization, also plays a pivotal role in determining insurance coverage. Our analysis reveals that households categorized as non-poor have a higher likelihood of being insured compared to those classified as moderately or severely poor. Additionally, the Ubudehe categories show a strong gradient: individuals in higher categories exhibit significantly higher odds of having insurance, with aORs ranging from 1.80 to 7.20. This correlation underscores the broader issue of socio-economic inequality, where wealthier households have the financial means to afford insurance premiums and access quality healthcare services. Conversely, those in lower socio-economic brackets often face financial constraints that limit their ability to enroll in insurance schemes. These findings suggest that policy interventions aimed at reducing poverty and providing financial subsidies or support for vulnerable populations could be effective in increasing insurance coverage. Geographic location is yet another determinant influencing health insurance uptake. The study found that residents of Kigali city are 1.50 times more likely to have health insurance compared to those living in other regions. This urban-rural divide may be attributable to several factors. Urban areas typically offer better access to information about health insurance options and more extensive healthcare infrastructure. Moreover, urban residents may have more stable employment and higher incomes, which facilitate the purchase of insurance. In contrast, rural populations may experience barriers such as limited access to healthcare facilities, lower income levels, and a lack of awareness about insurance benefits. Bridging this gap will likely require targeted outreach programs, improved healthcare infrastructure in rural areas, and tailored policies that address the unique challenges faced by rural communities.

Marital status also emerged as a significant factor in our study. The findings indicate that widowed household heads are more likely to have health insurance compared to single individuals, with an aOR of 1.20 for widows and 0.60 for singles relative to married individuals. These results might reflect the influence of social support networks, as married individuals or those with strong family ties may benefit from shared resources and information, which facilitate insurance enrollment. In contrast, single individuals might lack these support systems, thereby reducing their likelihood of obtaining insurance. This observation calls for the development of community-based interventions that specifically target single individuals to improve their access to health insurance. In synthesizing these findings, it becomes evident that enhancing health insurance coverage in Rwanda requires a multifaceted approach. First, addressing gender disparities is essential. Interventions should aim not only to promote health insurance among women who already exhibit higher uptake but also to encourage men to recognize the benefits of coverage. Public awareness campaigns, possibly integrated into workplace programs or community centers, could serve this purpose effectively. Second, educational initiatives are paramount. Improving health literacy can have a profound impact on insurance uptake, as more educated individuals are likely to make informed decisions regarding their health and financial well-being. Policymakers and stakeholders should consider incorporating insurance education into broader adult education and community outreach programs, particularly in areas with low literacy rates. Third, socio-economic factors cannot be ignored. Given that individuals from lower income brackets are less likely to be insured, governments and insurance providers should consider implementing sliding-scale premium structures or offering subsidies to make insurance more affordable for the economically disadvantaged. Additionally, improving the socio-economic status of vulnerable populations through comprehensive poverty reduction strategies will indirectly promote higher insurance uptake.

Furthermore, geographic disparities must be addressed by improving healthcare accessibility in rural areas. Investment in rural healthcare infrastructure, coupled with targeted information campaigns, can help bridge the urban-rural gap in insurance coverage. Mobile health units, community health workers, and telemedicine initiatives could also play crucial roles in reaching remote populations. Finally, marital status and social support systems should be considered when designing interventions. Tailored programs that foster community engagement and support for single individuals may help mitigate the lower insurance uptake observed in this group. Programs that encourage peer support and community-driven health initiatives can foster a culture where insurance is viewed as a collective good rather than an individual burden. The implications of our study are significant for policymakers. To achieve universal health coverage, Rwanda must implement targeted strategies that address these identified determinants. Future policies should prioritize increasing awareness about the benefits of health insurance, particularly among men and individuals with lower education levels, while also making insurance more affordable for low-income households. Additionally, expanding healthcare infrastructure and outreach in rural areas will be critical to ensuring that all Rwandans have equitable access to essential healthcare services. It is also essential for future research to explore these dynamics further. Longitudinal studies that track changes in insurance uptake over time, as well as qualitative research that delves into individual perceptions and barriers, would provide valuable insights to inform policy decisions. Moreover, comparative studies across different regions and socio-economic groups could help identify best practices and successful intervention models. Our study underscores the complex interplay of gender, education, socio-economic status, geographic location, and marital status in determining health insurance uptake in Rwanda. These findings are consistent with previous research conducted in similar contexts and highlight the need for a coordinated, multi-pronged policy approach. By addressing these factors comprehensively, Rwanda can move closer to achieving universal health coverage and ensuring that no individual is left without essential healthcare services.

## Conclusion

This study highlights the key socio-economic, demographic, and geographic factors influencing health insurance uptake in Rwanda. Among heads of households, 75.25% have health insurance, but significant disparities exist by gender, education, wealth, and location. Female heads of households, individuals with higher education, wealthier households, and those in urban areas, particularly Kigali, are more likely to be insured. These findings underscore the need for targeted interventions, such as health education programs, financial subsidies for low-income households, and improved outreach in rural areas to address gaps in insurance coverage. For policymakers, the study suggests that achieving universal health coverage requires strategies that promote awareness about the benefits of health insurance, particularly among men and those with lower education levels. Financial support for low-income populations and investments in rural healthcare infrastructure are critical. A coordinated approach can help Rwanda expand health insurance coverage and ensure equitable access to healthcare for all citizens.

### What is known about this topic

- Socio-economic and demographic factors significantly impact health insurance uptake, with education and income being crucial determinants;
- Barriers to health insurance enrollment include limited awareness, affordability issues, and disparities in access, particularly in low- and middle-income countries;
- Studies in Rwanda and Nigeria have identified similar trends, emphasizing the need for targeted interventions to improve health insurance coverage.

### What this study adds

- This study shows that females are 1.2 times more likely to be insured than males, highlighting the importance of gender-focused strategies in health insurance promotion;
- It reveals that household heads aged 14-24 and those in the lowest wealth quintile are significantly less likely to be insured, emphasizing the need for policies tailored to these demographics;
- The findings indicate that households in Kigali are 1.5 times more likely to have health insurance, stressing the importance of addressing regional disparities in insurance access.
